# Overexpression of CTHRC1 in Hepatocellular Carcinoma Promotes Tumor Invasion and Predicts Poor Prognosis

**DOI:** 10.1371/journal.pone.0070324

**Published:** 2013-07-29

**Authors:** Yu-Ling Chen, Ting-Huang Wang, Hey-Chi Hsu, Ray-Hwang Yuan, Yung-Ming Jeng

**Affiliations:** 1 Graduate Institute of Pathology, National Taiwan University, Taipei, Taiwan; 2 Department of Pathology, National Taiwan University Hospital, Taipei, Taiwan; 3 Department of Surgery, National Taiwan University Hospital, Taipei, Taiwan; University of Hong Kong, Hong Kong

## Abstract

Collagen triple helix repeat containing-1 (CTHRC1) is a secreted glycoprotein that activates the planar cell polarity pathway of Wnt signaling. Using microarray analysis, we found that the *CTHRC1* gene is overexpressed in hepatocellular carcinoma (HCC). The level of *CTHRC1* mRNA was measured in 201 surgically resected HCCs using real time reverse transcription-polymerase chain reaction. Overexpression of CTHRC1 in HCC was associated with large tumor size and advanced tumor stage. Furthermore, expression of CTHRC1 as was identified as an independent prognostic factors in the multivariate analysis. Suppression of CTHRC1 expression inhibited tumor migration and invasion whereas overexpression of CTHRC1 promoted tumor invasion. Activation of RhoA, but not Rac1 or Cdc42, was found to play a crucial role in CTHRC1-induced cell migration. CTHRC1 promoted adhesion of cancer cells to extracellular matrix through induction of integrin β1 expression and activation of focal adhesion kinase. These results suggest CTHRC1 promotes tumor invasion and metastasis by enhancing the adhesion and migratory abilities of tumor cells. It is also a promising biomarker for predicting the prognosis of patients with HCC.

## Introduction

Hepatocellular carcinoma (HCC) is one of the most common fatal malignancies in Taiwan and many other countries in Asia and Africa [1]. Its incidence is increasing in Western countries, mainly due to the prevalence of chronic hepatitis C infection [2]. The major risk factors are hepatitis B and hepatitis C infections, cirrhosis of any etiology, and aflatoxin exposure [3]. Molecular approaches have revealed the involvement of *p53* and *β-catenin* mutations in hepatocarcinogenesis [4], [5]. The mutations account for approximately 50% of all HCC cases. Somatic mutations of other known oncogenes and tumor suppressor genes in HCC are rare. Hence, the molecular mechanisms of HCC, particularly in the early stage, remain largely unclear.

Collagen triple helix repeat containing-1 (CTHRC1) is a 25-kDa secreted glycoprotein containing an NH_2_-terminal peptide for extracellular secretion, a short collagen triple helix repeat of 36 amino acids, and a COOH-terminal globular domain [6]. It was initially found in a screen for differentially expressed genes in balloon-injured versus normal rat arteries [6]. Expression of Cthrc1 was induced abundantly in adventitial fibroblasts and neointimal smooth muscle cells of balloon-injured vessels [6]. Forced expression of Cthrc1 in rat fibroblasts promoted cell migration and inhibited collagen I synthesis in these cells, indicating Cthrc1 may contribute to tissue repair in vascular remodeling in response to injury by limiting collagen matrix deposition and promoting cell migration [6]. Likewise, Cthrc1 transgenic mice showed reduced neointimal lesion formation and adventitial collagen deposition in response to carotid artery ligation [7]. The molecular mechanisms of Cthrc1 are yet to be discovered. In smooth muscle of arteries, Cthrc1 inhibits the signaling of transforming growth factor-β [7]. Recently, Cthrc1 was found to selectively activate the planar cell polarity pathway of Wnt signaling by stabilizing the Wnt-receptor complex [8].

The human homolog of CTHRC1 shares 92% sequence identity with the rat protein. CTHRC1 was found to be expressed in 16 out of 19 types of human solid tumors including invasive melanoma but not in benign nevus and non-invasive melanoma [9]. However, the functional role of CTHRC1 in HCC and other cancers is still uncharacterized. In the present report, we show that CTHRC1 plays an important role in migration and invasion of hepatocellular carcinoma and that overexpression of CTHRC1 predicts poor prognosis in patients with HCC.

## Materials and Methods

### Ethics Statement

All experimental procedures were undertaken at the National Taiwan University Hospital. The study was undertaken on project license number 201107042RC which was approved by the Ethics Committee of the National Taiwan University Hospital. The live participants had provided written informed consents. In addition, the written informed consent was obtained from the parents on the behalf of a teenager participant involved in our study. The need for written informed consent from the deceased participants and the next of kin of the deceased participants was waived by the Ethics Committee of the National Taiwan University Hospital.

### Liver Samples

From 1982 to 1998, unifocal, primary HCCs surgically resected from 201 patients who received detailed pathological assessment and regular follow-up at the National Taiwan University Hospital were selected for this study. All the specimens were rendered anonymous and evaluated in a blinded manner. The patients included 157 men and 44 women with a mean age of 55.1 years (range, 8–88). Serum hepatitis B surface antigen (HBsAg) was detected in 142 cases and anti-hepatitis C (HCV) antibody in 56, including 18 positive for both. Liver cirrhosis was found in 88 cases (43.8%). None had received transhepatic arterial embolization or chemotherapy before tumor resection. After surgery, all patients received laboratory examinations such as serum α-fetoprotein (AFP) at 1–6 month intervals, and ultrasonography of liver at 3–12 month intervals. In all, 154 patients died within 10 years. Follow-up periods for survivors were 27–248 months (median, 142).

### Histology Study and Tumor Staging

Surgically resected specimens were formalin-fixed, paraffin embedded, and cut into sections (5-µm thick). The sections were stained with hematoxylin and eosin and reviewed by one of the authors (H.C. H.) to determine tumor grade and stage. The tumor grade was based on the criteria proposed by Edmonson and Steiner [10]. The tumors were staged according to American Joint Commission on Cancer system [11]. The margins of surgical specimens were inked and checked under a microscope. Only completely resected specimens were included in this study.

### Microarray Analysis

Total RNA was isolated from paired specimens of tumor and non-tumorous liver parenchyma using TRIzol (Life Technologies, Invitrogen, Carlsbad, CA) according to the manufacturer’s instructions. The complementary DNA was labeled with Cy3-dCTP or Cy5-dCTP, and then applied to a microarray chip. The microarray experiment and data analysis were done by Welgene Biotech Company (Taipei, Taiwan) using the Agilent Oligo Chip (Human 1A Oligo Chip V2). Microarrays were scanned by laser scanner and the microarray signal intensities were measured to identify gene expression differences. The microarray data were deposited in the Gene expression Omnibus database (GEO accession number: GSE46408).

### RNA Isolation and Real Time RT-PCR

Total RNA was isolated from tissue samples and cell lines using the Trizol reagent. The SYBR green-based real time reverse transcription-polymerase chain reaction (RT-PCR) assay was used to determine the mRNA level of *CTHRC1* in the paired HCC and non-tumorous liver tissue samples using the ABI PRISM 7900HT Sequence Detection System (Applied Biosystems, Foster City, CA). Glyceraldehyde-3-phosphate dehydrogenase (GAPDH), a housekeeping gene, served as a control for RNA quantity. The primers for CTHRC1 were CTHRC1-F (5′-GCATGCTGTCAGCGTTGGTA-3′) and CTHRC1-R (5′-TCAATGGGAAGAGGTCCTGAA-3′). The primers used for GAPDH were GAPDH-F (5′-AGCCTCAAGATCATCAGCAATGCC-3′) and GAPDH-R (5′-TGTGGTCATGAGTCCTTCCACGAT-3′). In a volume of 20 µl PCR reaction, 1 µl of complementary DNA template was mixed with 10 µl of 2× Power SYBR® PCR master mix (Applied Biosystems), 200 nM of paired primers, and distilled water. PCR amplification included initial incubation at 50°C for 2 min, denaturing at 95°C for 10 min, and 40 cycles of denaturing at 95°C for 15 s and annealing at 60°C for 1 min. Receiver operating characteristic (ROC) analysis was used to select the optimal cut-off point for statistical analysis.

### Cell Culture and Treatment

Huh7 and 293T cells were purchased from the American Type Culture Collection. GP2-293 cells were purchased from Clontech Inc. HA22T cells were purchased from Bioresource Collection and Research Center of Taiwan. All the cells were grown in DMEM with 10% fetal bovine serum. All the cells were grown at 37°C in a humidified atmosphere composed of 95% air and 5% CO_2_. The cells were passaged with trypsin/EDTA at 80?90% confluence. Untagged recombinant human CTHRC1 protein generated by wheat-germ cell free protein expression system was purchased from Abnova (Taipei, Taiwan). For the inhibition of ROCK activity, Huh7 cells were pretreated with a ROCK inhibitor Y27623 (5 µmol/L) for 1 h, and then treated with recombinant CTHRC1 (100 ng/ml) for 30 min.

### Plasmid, Transfection, and Retroviral Infection

The open reading frame of *CTHRC1* was amplified from HeLa cells by RT-PCR using the primers: CTHRC1-F: ATAAGAATGCGGCCGCTCCACCATGCGACCCCA and CTHRC1-R: GCGTCGACTTTTGGTAGTTCTTCAATAATG. The PCR products were cloned to pCMV-Tag 4A (Stratagene, La Jolla, CA) and subcloned into a retroviral expression vector pQCXIP (Clontech, Mountain View, CA) to generate pQCXIP-CTHRC1. pQCXIP-CTHRC1 was cotransfected with pVSV-G (Clontech) into the retroviral package cell GP2-293 to produce retrovirus. After incubating with medium containing retroviral particles and 8 µg/ml polybrene (Sigma-Aldrich, St. Louis, MO) for two days, the target cells were treated with puromycin (2 µg/ml) (Clontech) for two weeks to select the cells with stably integrated retroviral vectors.

### RNA Interference

To knockdown the expression of CTHRC1, CTHRC1 shRNA in a lentiviral plasmid and control LacZ shRNA were purchased from the National RNAi Core Facility (Academia Sinica, Taipei, Taiwan). The target sequences were as follows: shCTHRC1-1: GCGTTGGTATTTCACATTC; shCTHRC1-2: CGGGATGGATTCAAAGGAG; shCTHRC1-3: CGCATCATTATTGAAGAAC; shCTHRC1-4: CCTGTATAATGGAATGTGC; shCTHRC1-5: GTGAAGGAATTGGTGCTGG. The lentivirus packaging, infection, and selection were performed as previously described [12].

### Generation of Mouse Polyclonal Antibodies Against CTHRC1 Protein

The full-length open reading frame of *CTHRC1* was amplified by PCR and cloned into expression vector pET32a (Merck, Darmstadt, Germany). The recombinant His-tagged CTHRC1 protein produced from *Escherichia coli* was purified on a Ni Sepharose 6 Fast Flow column (GE Healthcare, Uppsala, Sweden), injected subcutaneously into mice, and boosted for three times to generate polyclonal antibodies.

### Western Blot

Protein samples (60 µg each) were separated by 10% SDS-PAGE and then electrotransferred onto nitrocellulose membranes (Amersham, Buckinghamshire, UK). The membranes were then exposed to primary and secondary antibodies at optimum dilution, and the immunoreactive signals detected with Immobilon Western Chemiluminescent HRP Substrate (Millipore, Bedford, MA). The primary antibodies were to CTHRC1 (1∶1000; home-made, mouse polyclonal), phosphorylated FAK at Tyr576/577(p-FAK^576/577^; 1∶1000; Cell Signaling, Beverly, MA), phosphorylated FAK at Tyr397(p-FAK^397^; 1∶1000; Cell Signaling, Beverly, MA), FAK (1∶2000; Cell Signaling), RhoA (1∶1500; Cell Signaling), Rac1 (1∶1000; Cell Signaling), Cdc42 (1∶1000; Cell Signaling), Myosin light chain 2 (MLC2) (1∶1000; Cell Signaling), phosphorylated MLC2 at Thr18/Ser19 (p-MLC2; 1∶1000; Cell Signaling), integrin β1 (1∶2000; Cell Signaling), and α-actin (1∶4000; Sigma-Aldrich).

### Detection of CTHRC1 in Culture Medium

The proteins in culture medium were concentrated by the methanol/chloroform protein precipitation method [13]. Briefly, 800 µl of methanol followed by 200 µl of chloroform were added to 200 µl culture medium and mixed thoroughly by vortexing. Then 600 µl of ultrapure water was added and mixed well by vortexing. After centrifuging at 14,000 × g for 5 min, the upper aqueous layer was removed. 600 µl of methanol was added and mixed by vortexing. After pelleting the proteins by centrifuging at 14,000 × g for 5 min, the methanol/chloroform mixture was removed. The pellets were dissolved in loading buffer, and the protein levels were determined by western blotting.

### Cell Proliferation Assay

Cell survival and proliferation was measured using MTT (3-[4, 5-dimethylthiazol-2-yl]-2, 5-diphenyl-2H-tetrazolium bromide) assay. Cells (2000 per well) were seeded into 96-well plates, and incubated at 37°C in a humidified atmosphere with 5% CO_2_. At the appropriate time interval, MTT (2 mg/ml in PBS) was added and incubated for 4 h. The resulted color reaction product, MTT formazan, was extracted with dimethyl sulfoxide and the absorbance at 570 nm measured using a microplate reader.

### Tumorigenicity Assay

NOD/SCID mice (female, 4–6 weeks old) were purchased from the National Taiwan University Laboratory Animal Center and accommodated for 7 days for environmental adjustment prior to experimentation. Cells were trypsinized, resuspended in serum-free DMEM, and injected subcutaneously (2×10^6^ cells in a total volume 0.1 ml) into both flanks. Animals were observed weekly for tumour development for 5 to 8 weeks. The final tumor weights at the time of animal sacrifice were recorded. The animal experiments were approved by the Committee on Animal Research of National Taiwan University.

### Boyden Chamber Assay

For invasion assays, we used modified Boyden chambers with filter inserts (pore size, 8 µm) coated with Matrigel (30 µg; Collaborative Biomedical Products, Bedford, MA) in 24-well tissue culture plates (Nucleopore, Pleasanton, CA). Cells (2×10^4^) in 100 µl of serum-free DMEM were placed in the upper chamber, and 0.5 ml of DMEM containing 10% FBS was placed in the lower chamber. After 24 h in culture, cells were fixed in 3.7% formaldehyde in PBS for 15 min and then stained with 4′-6-diamidino-2- phenylindole (DAPI) in 0.1% Triton X-100 containing PBS for 15 min. Cells on the upper side of the filters were removed with cotton-tipped swabs, and the filters were washed with PBS. Cells on the underside of the filters were viewed and counted under a fluorescence microscope. Each group was plated in triplicate in each experiment, and each experiment was repeated three times. The cell motility assay was done in the same way as invasion assay except the filters were not coated with Matrigel and the incubation time was 20 h.

### Scratch Wound Assay

HA22T cells were grown to confluence in six-well culture plates. Cell layers were scraped with a sterile pipette tip and reincubated at 37°C. Photographs were taken at indicated time points. Migration from the edge of the injured monolayer was quantified by measurement of the distance between the wound edges. Experiments were done in triplicate and repeated twice.

### Adhesion Assay

Cells (1×10^4^ cells per well) were seeded onto 96-well plates coated with 20 µg/ml fibronectin (Sigma-Aldrich). After incubated at 37°C for the indicated period, the cells were washed with PBS 3 times, fixed with 3.7% formaldehyde, and stained with crystal violet. The adherent cells were counted under a microscope.

### Immunofluorescence Staining

Cells were trypsinized, replated onto 20 µg/ml fibronectin coated slides, and incubated at 37°C for 60 min, Cells were fixed with 4% paraformaldehyde in PBS for 10 min, permeabilized in 0.5% Triton X-100 at room temperature for 5 min, and blocked with blocking solution (0.5% FBS and 0.1% Triton X-100 in PBS). Cells were stained with mouse anti-vinculin antibody (1∶400; Thermo Fisher Scientific, Waltham, MA) in blocking solution for 2 h, washed with PBS 3 times, and then incubated with fluorescein isothiocyanate (FITC)-conjugated goat anti-mouse secondary antibody (1∶1000; Alexa Fluor 488; Life Technologies) and rhodamine-phalloidin (1∶500; Life Technologies) for 1 h. Cells were washed, mounted, and observed under a fluorescence microscope.

### 3D Collagen Gel Invasion Assay

Cells (10^4^ per well) were resuspended in collagen gel mixture (70 µl of 3 mg/ml rat tail collagen I, 9 µl of 10x DMEM, 2 µl of 0.2 M HEPES pH 7.3, 8 µl of H_2_O, with the pH value adjusted to around 7.4 with 5 N NaOH) and seeded onto 96-well plates. The gel was incubated 30 min at 37°C to solidify, and then supplemented with 100 µl of DMEM containing 1% FBS, incubated 3 days, observed for change in cell-spreading phenotype, and photographed using an inverted photomicroscope.

### Rho Protein Activation Assay

RhoA, Rac1, and Cdc42 activities in cells were measured using Rho protein activation assay kits (Cytoskeleton, Denver, CO) according to the manufacturer’s instructions. CTHRC1-knocked down HA22T cells were seeded in 100-mm dishes, cultured to 40–60% confluence, and serum-starved overnight. After stimulated with 10% FBS for 30 min at 37°C, the cells are washed with ice cold PBS, and lysed with cell lysis buffer. Lysates were harvested by centrifugation at 10,000 rpm for 2 min and assayed to determine protein concentration using a protein assay kit (BioRad, Richmond, CA). In pull-down assays, equal amounts of protein from cell lysates were incubated for 2 h at 4°C with 50 µg of Rhotekin-RBD or PAK-PBD protein agarose beads. Pellets were washed and subjected to western blotting using anti-RhoA, rabbit anti-Rac1, and Cdc42 antibodies.

### Statistical Analysis

The data analyses were carried out using MedCalc (MedCalc Software, Mariakerke, Belgium) software. Correlation between CTHRC1 expression and clinicopathologic parameters was evaluated using the χ^2^ test. Multivariant analyses were conducted by multiple Cox’s proportional hazards models Survival rates were calculated using the Kaplan-Meier method, and difference in survival curves was analyzed by using the log-rank test. Student’s t-test was used for comparisons of cell number, tumor weights, and luciferase values. Two-tailed *P*<0.05 was considered statistically significant.

## Results

### CTHRC1 is Overexpressed in HCC

**Figure 1:**
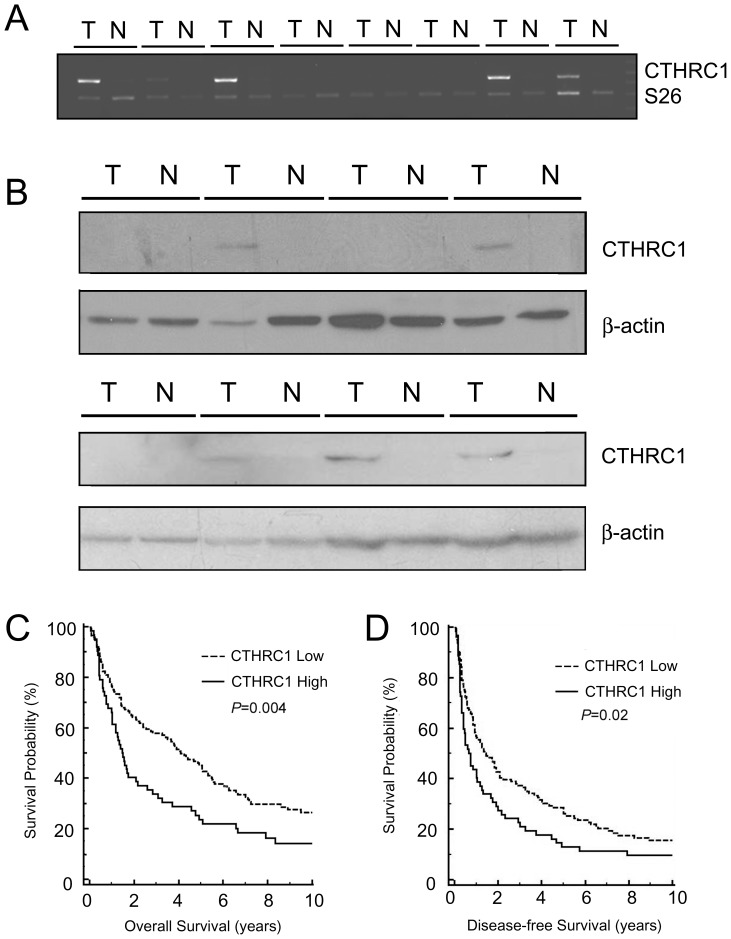
Overexpression of CTHRC1 in HCC. (A) RT-PCR analysis showed overexpression of CTHRC1 in 4 of 8 HCC specimens (T), but in none of the 8 specimens of nontumorous liver parenchyma (N). S26 (ribosomal protein S26) served as internal control. (B) Western blotting showed CTHRC1 was expressed in 5 of 8 HCC specimens (T) but not in nontumourous liver parenchyma (N). (C, D) Cumulative overall (C) and disease-free (D) survival curves for 201 patients with resected, unifocal, primary HCC stratified by *CTHRC1* mRNA expression level as determined by real time RT-PCR. Patients with HCCs overexpressing the *CTHRC1* gene had significantly lower 10-year overall and disease-free survival rates than those with HCCs without *CTHRC1* overexpression.

Using microarray analysis for six pairs of HCC and corresponding nontumorous liver parenchyma, we found the *CTHRC1* gene was overexpressed in three of the six HCCs. Semi-quantitative RT-PCR was employed for measurement of *CTHRC1* mRNA expression in paired HCC and nontumorous liver parenchyma and showed overexpression of *CTHRC1* in 4 of 8 HCCs (Figure 1A). To further confirm that CTHRC1 is overexpressed in HCC, the protein levels of CTHRC1 in HCC tissue samples were analyzed by western blotting. As shown in Figure 1B, CTHRC1 was overexpressed in 5 of the 8 HCC specimens, but not in nontumourous liver parenchyma. To determine the clinicopathological significance of CTHRC1 expression, 201 unifocal HCCs were analyzed by quantitative RT-PCR. An ROC curve demonstrated that ΔCt value of 8.48 was the optimal cutoff point for predicting patient death within four years after operation. The area under curve (AUC) was 0.621 (Figure S1). Therefore, we segregated the tumors into CTHRC1-high (ΔCt <8.48) and CTHRC1-low (ΔCt ≥8.48) groups. Using this cutoff point, CTHRC1 was overexpressed (ΔCt <8.48) in 62 tumors (30.8%). In contrast, none of the 103 paired non-tumorous liver RNA samples available for examination overexpressed CTHRC1.

To elucidate the role of CTHRC1 in HCC progression, we evaluated the correlation of CTHRC1 expression with a variety of clinicopathologic factors. As shown in Table 1, CTHRC1 expression correlated with large tumor size (>5 cm; *P* = 0.008) but not with sex, age, HBsAg status, α-fetoprotein (AFP) level, tumor grade, *p53* mutation, and *β-catenin* mutation. Importantly, CTHRC1 was expressed much more frequently in advanced stage (stage II-III) HCCs than in early-stage HCCs (stage I) (*P* = 0.014). Compared to those with CTHRC1-low HCCs, patients with CTHRC1-high HCCs had a lower 10-year overall and disease-free survival rates (Figure 1C and D).

**Table 1:** Correlation of CTHRC1 expression and clinicopathological factors.

		CTHRC1		Odds ratio	*P* value
		+ (n = 62)	− (n = 139)	(95% CI)	
Age (year)	< = 55	30	59	1.27(0.67–2.42)	NS
	>55	32	80		
Sex	Male	48	109	0.94(0.43–2.06)	NS
	Female	14	30		
HBsAg	(−)	17	34	1.11(0.53–2.32)	NS
	(+)	44	98		
AFP (ng/mL)	<200	25	72	0.56(0.29–1.09)	NS
	> = 200	37	60		
Size (cm)	< = 5	21	75	0.44(0.22–0.85)	0.008
	>5	41	64		
Grade	1–2	33	93	0.56(0.29–1.09)	NS
	3–4	29	46		
Stage	I	18	66	0.45(0.23-0.9)	0.014
	II–III	44	73		
*p53* mutation	(−)	20	46	0.67(0.31–1.46)	NS
	(+)	26	40		
*β-catenin* mutation	(−)	45	79	3.23(0.84–14.7)	NS
	(+)	3	17		

Among those parameters analyzed, high AFP level, large tumor size, high tumor grade, high tumor stage, CTHRC1 overexpression, *p53* mutation, and absence of *β-catenin* mutation were significantly associated with poor prognosis in univariat analysis (Table 2). In multivariate analysis of prognostic factors, only tumor stage and CTHRC1 expression were significant and independent prognostic factors for HCC patients (Table 3).

**Table 2:** Univariate analysis of prognostic factors in patients with HCCs.

Variables	Hazards ration	95%CI	Unfavorable/Favorable	*P*-value
Age (years)	1.049	0.754–1.457	>55/< = 55	0.7776
Sex	1.134	0.772–1.666	Male/Female	0.5209
Size	2.023	1.446–2.831	>5 cm/< = 5 cm	0.0001*
AFP (ng/mL)	1.645	1.182–2.289	> = 200/<200	0.0032*
CTHRC1	1.632	1.160–2.296	High/Low	0.0049*
Grade	1.505	1.081–2.096-	3,4/1,2	0.0152*
Stage	2.950	2.073–5.198	II–III/I	<0.0001*
*p53* mutation	1.938	1.320–2.846	Positive/Negative	0.0007*
β-catenin mutation	2.097	1.206–3.697	Negative/Positive	0.0089*

* *P*<0.05.

**Table 3:** Multivariate analysis of prognostic factors in patients with HCCs.

	Variables	Hazards ration	95%CI	Unfavorable/Favorable	*P*-value
Multivariate analysis	Size	1.0782	0.706–1.647	>5 cm/< = 5 cm	0.6070
	AFP (ng/mL)	1.104	0.722–1.690	> = 200/<200	0.6454
	CTHRC1	1.644	1.085–2.490	High/Low	0.0189*
	Grade	1.232	0.825–1.838	3,4/1,2	0.3077
	Stage	2.717	1.671–4.417	II–III/I	0.0001*
	*p53* mutation	1.516	0.997–2.306	Positive/Negative	0.0517
	β-catenin mutation	1.043	0.549–1.982	Negative/Positive	0.8969

* *P*<0.05.

### CTHRC1 Promotes Tumor Migration and Invasion

**Figure 2:**
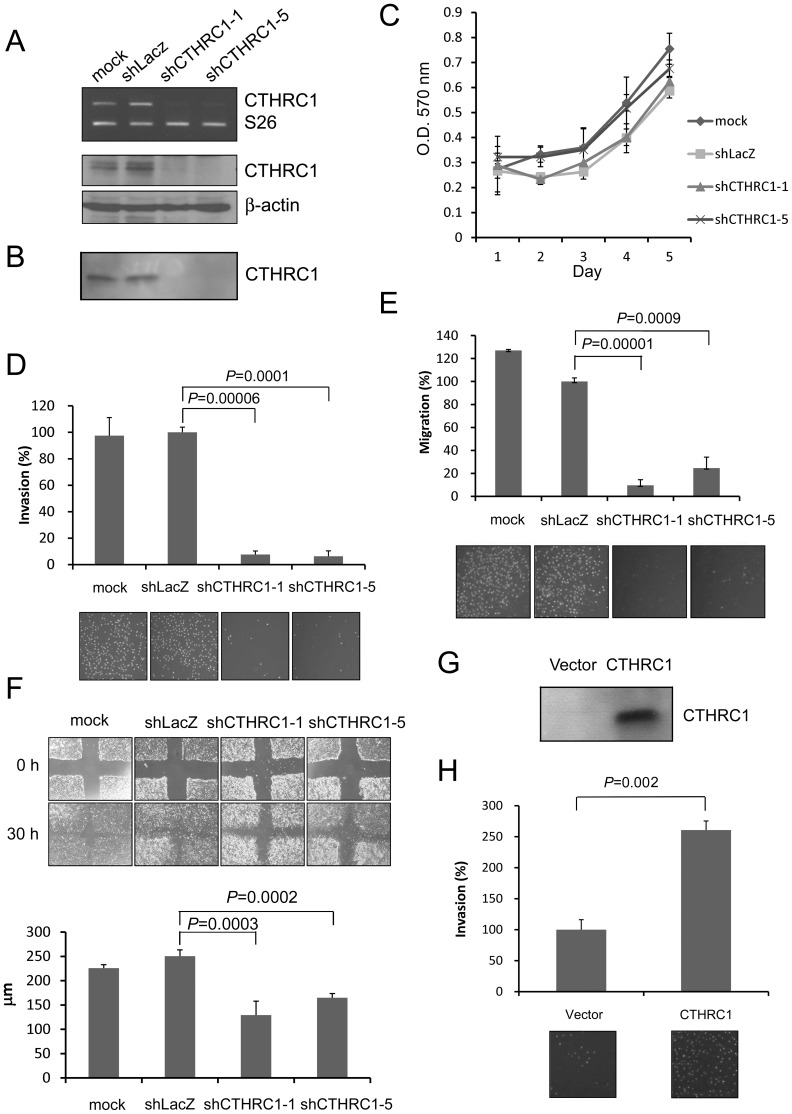
CTHRC1 promotes tumor migration and invasion. (A) shRNA#1 and #5 markedly reduced CTHRC1 mRNA and protein in HA22T cells. (B) Knockdown of CTHRC1 markedly decreased the amount of CTHRC1 in the culture medium. (C) Depletion of CTHRC1 did not affect proliferation, as measured by MTT assay. (D–E) Knockdown of CTHRC1 in HA22T cells inhibited invasion (D) through Matrigel and motility (E). (F) Scratch wound assay. Knockdown of CTHRC1 in HA22T cells delayed closure of scratch wounds Photographs were taken at 30 h. (G) Overexpression of CTHRC1 in Huh7 increased the amount of CTHRC1 in the culture medium. (H) Overexpression of CTHRC1 in Huh7 cells enhanced tumor invasion through Matrigel.

In HCC, advanced stage is characterized by vascular invasion and various degrees of intrahepatic metastasis. Since CTHRC1 overexpression in HCC is associated with advanced stage, we speculated that CTHRC1 may play a role in tumor growth and invasion. To test this hypothesis, we evaluated the expression of CTHRC1 in HCC cell lines and found that HA22T and Huh7 cells had the highest and lowest CTHRC1 expression, respectively. So we selected them for further study. Five different lentiviral constructs carrying CTHRC1 shRNAs were used to transduce HCC cell line HA22T. Cells transduced with shRNAs #1and #5 resulted in a dramatic reduction of CTHRC1 mRNA and protein expression (Figure 2A) and the amount of CTHRC1 in the culture medium (Figure 2B) and were selected for subsequent studies.

We first tested the effect of CTHRC1 knockdown on cell proliferation. MTT assay showed that knockdown of CTHRC1 had no effect on cell survival and proliferation (Figure 2C). When injected into NOD/SCID mice, HA22T cells with or without CTHRC1 knockdown cannot form tumor up to 8 weeks after injection (data not shown). Overexpression of CTHRC1 in Huh7 cells did not enhance tumor growth in NOD/SCID mice (Figure S2). These results indicate that CTHRC1 does not promote growth of HCC.

**Figure 3:**
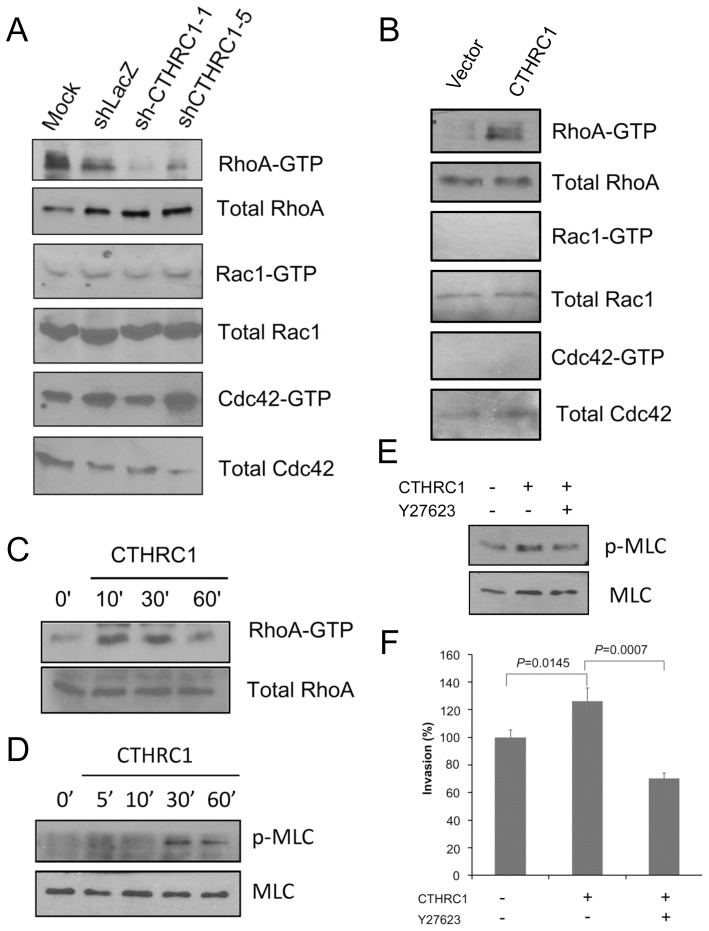
CTHRC1 activates RhoA activity. (A) Pull-down assay of Rho family proteins showed that knockdown of CTHRC1 inhibited the activation of RhoA, but not Rac1 or Cdc42. (B) Treating Huh7 cells with recombinant CTHRC1 (100 ng/ml) activated the RhoA pathway. (C) CTHRC1 enhanced phosphorylation of MLC2, a target of ROCK. (D) Pretreatment with a ROCK inhibitor Y2732 (5 µmol/L) abolished the invasion-promoting activity of CTHRC1.

In the modified Boyden chamber assay, knockdown of CTHRC1 dramatically inhibited tumor invasion (Figure 2D). To elucidate the mechanism of this inhibition, a transwell migration assay was performed and showed similar reduction in the number of migrating cells, indicating the reduction in invasive capacity is mainly due to reduced cell migration (Figure 2E). Scratch wound assay also showed that closure of the wound was delayed in CTHRC1-knocked down HA22T cells, further confirming the effect of CTHRC1 on tumor cell migration (Figure 2F). On the other hand, when CTHRC1 was overexpressed in Huh7 cells by retroviral transduction (Figure 2G), invasiveness of the tumor cells was enhanced (Figure 2H). To confirm the role of CTHRC1 in tumor invasion, cells were grown in a 3-D gel consisting of a thick matrix of native collagen type I fibers. After incubation for 3-days, multiple long protrusions could be seen in parental cells and control shRNA transduced cells but not in CTHRC1-knocked down cells (Figure 3).

### CTHRC1 Promotes Cell Migration by Activation of RhoA

The Rho family of small GTPase regulates cell motility by orchestrating reorganization of the cytoskeleton [14]. Their participation in the process of tumor (e.g., HCC) invasion and metastasis has been suggested [15]. Therefore, it was of interest to determine whether GTP-binding proteins of the Rho family are involved in CTHRC1-mediated invasion of HCC cells. As shown in Figure 3A, knockdown of CTHRC1 decreased the activity of RhoA (relative amount of RhoA in the active GTP-binding form) but not of the two other Rho-family small GTPases, Rac1 and Cdc42, in HA22T cells. On the other hand, overexpression of CTHRC1 in Huh7 cells increased the activity of RhoA but not that of Rac1 and Cdc42 (Figure 3B). Treating Huh7 cells with recombinant CTHRC1 also enhanced RhoA activity (Figure 3C). ROCK is a kinase associated with and activated by GTP-bound form of RhoA for transducing RhoA signaling [16]. CTHRC1 treatments induced phosphorylation of MLC2, a ROCK target (Figure 3D). The enhanced phosphorylation of MLC2 by CTHRC1 was abolished by 5 µmol/L Y27632, a ROCK inhibitor (Figure 3E). The invasion-promoting activity of CTHRC1 was inhibited by Y27632 treatment (Figure 3F). Taken together, these data suggest that CTHRC1 promotes tumor invasion by activating RhoA/ROCK pathway.

### CTHRC1 Promotes Cell-matrix Adhesion by Enhancing Integrin β1 Expression

**Figure 4:**
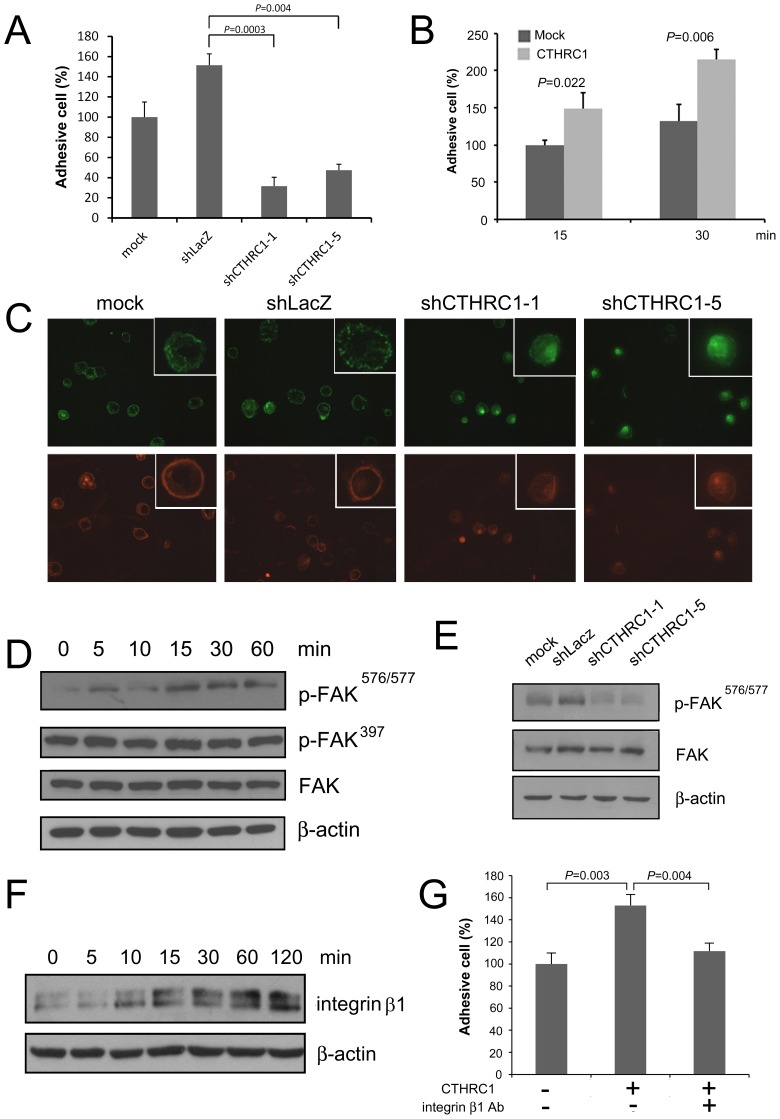
CTHRC1 promotes cell adhesion through activation of integrin β1/FAK pathway. (A) Adhesion assay. Approximately 1×10^4^ cells were seeded into fibronectin-coated 96-well plates and incubated at 37°C for 30 min, washed, fixed, and stained with crystal violet. The remaining cells were counted. Knockdown of CTHRC1 markedly reduced the number of cells adherent to fibronectin. (B) Treating Huh7 cells with CTHRC1 (100 ng/ml) enhanced adhesion to fibronectin at 15 min and 30 min. (C) Knockdown of CTHRC1 decreased the formation of focal adhesion structures. Cells were trypsinized and replated onto fibronectin-coated slides and incubated at 37°C for 60 min, fixed, and stained. Mouse anti-vinculin was used to visualize focal adhesions (upper panel), and rhodamine-phalloidin was used to visualize actin filaments (lower panel). (D) CTHRC1 treatment enhanced phosphorylation of FAK at residues 576/577. (E) Knockdown of CTHRC1 inhibited phosphorylation of FAK. (F) CTHRC1 enhanced expression of integrin β1. (G) The adhesion-promoting effect of CTHRC1 was abolished by integrin β1 blocking antibody.

Cell-matrix adhesion is an important step in cell migration. So we compared the adhesive capability of HA22T cells before knockdown of CTHRC1 with that of the same cells after knockdown. Cells were detached with trypsin-EDTA and plated on fibronectin-coated coverslips in medium for 30 min. After washing, the adherent cells in five random fields were counted under a light microscope. As shown in Figure 4A, compared with the number of adhering parental cells and vector-control cells, the number of adherent CTHRC1 knocked-down cells was significantly decreased. Treating Huh7 cells with recombinant CTHRC1 enhanced adhesion to fibronectin (Figure 4B). Vinculin is a cytoskeletal protein that participates in cell adhesion and migration by linking the actin cytoskeleton to transmembrane receptors, integrin, and cadherin [17]. Immunofluorescence staining for vinculin after allowing the cells to adhere to fibronectin-coated coverslips for 60 min showed less spreading of cells with knockdown of CTHRC1 compared to control cells, and showed less well-developed focal adhesions (Figure 4C). Focal adhesion kinase (FAK), a nonreceptor tyrosine kinase, is an important positive regulator cell of spreading and migration [18]. FAK is activated by tyrosine autophosphorylation in response to integrin clustering achieved by cell adhesion [19]. As shown in Figure 4D and 4E, CTHRC1 induced phosphorylation of FAK at residues 576/577, whereas knockdown of CTHRC1 inhibited autophosphorylation of FAK.

Integrin β1 mediates adhesion of cells with fibronectin [20]. CTHRC1 enhanced the expression of integrin β1 in Huh7 cells (Figure 4F). The adhesion-promoting effect of CTHRC1 was abolished by integrin β1 blocking antibody (Figure 4G), indicating CTHRC1 promotes cell adhesion through induction of integrin β1.

## Discussion

Cancer invasion and metastasis are complex processes involving increase in cell motility, adhesion to underlying stroma, digestion of extracellular matrix, vascular invasion, evasion of immune attack, and growth in foreign environments. Many mechanisms regulate these processes. One way to identify the genes involved in these processes is to compare the gene expression profile between tumor and nontumorous tissue [12], [21]. In the present study, we used genome-wide microarray analysis and identified *CTHRC1* as another gene overexpressed in HCC. Overexpression of CTHRC1 was seen more frequently in larger and more advanced stage tumors. Besides, expression of CTHRC1 was an indicator of poor prognosis. These results suggest a functional role of CTHRC1 in tumor progression. Previous studies also found overexpression of CTHRC1 in melanoma and cancers of the gastrointestinal tract, lung, breast, ovary, and many other organs [9]). These observations indicate that CTHRC1 is commonly overexpressed in cancer.

*Cthrc1* was initially found in a screening for differentially expressed genes in balloon injured versus normal rat vessels [6]. Forced expression of Cthrc1 was found to promote migration of rat fibroblasts. So we speculated that cancer cells secrete CTHRC1 to promote their migration. Indeed, knockdown of CTHRC1 inhibited invasiveness of HCC cells in a transwell invasion assay. On the other hand, overexpression of CTHRC1 promoted tumor invasion. The enhanced invasiveness is mostly attributed to the increase in cell migration as demonstrated in the transwell migration assay and scratch wound assay. A similar promigratory role has been reported for melanoma [9].

It is still not clear how CTHRC1 regulates cancer cell migration. Pyagay et al. reported overexpression of Cthrc1 caused a dramatic reduction in the synthesis of collagen type I in rat fibroblasts [6], but we found no change in collagen synthesis in HA22T cells with knockdown of CTHRC1 (data not shown). So we decided to test the important signaling pathways involved in cell migration. The Rho family of small GTPase, including RhoA, Cdc42, and Rac1, are key regulators of cell migration based on their effects on the cytoskeletal reorganization and cell adhesion activities [15]. Therefore. we tested the activity of the Rho family proteins and found CTHRC1 specifically inhibits the activation of RhoA. Interestingly, CTHRC1 was reported to be an activator of the planar cell polarity pathway of Wnt signaling in mouse embryogenesis [8]. Planar cell polarity signaling regulates the establishment of polarity within the plane of the epithelium and allows cells to obtain directional information during embryogenesis [22]. One of the major components of the planar cell polarity pathway is the activation of small GTPases, including RhoA and Rac1, and their downstream protein kinases, ROCK and JNK, respectively, to regulate cytoskeletal reorganization [23]. Hence, by secreting CTHRC1 into its microenviroment, cancer cells stimulate their own motility and that of adjacent cancer cells by activating RhoA signaling.

Activation of RhoA mediates the formation of stress fibers and the integrin adhesion complex to anchor cells to their substrata [24], [25]. Adhesion to stroma is essential for cell viability and migration. Our study found that CTHRC1 enhanced the ability of HCC cells to adhere to fibronectin-coated slides. Induction of integrin β1 expression and activation of FAK phosphorylation to promote focal adhesion formation may account for this effect.

During preparation of this manuscript, Park *et al.* reported that CTHRC1 is overexpression in pancreatic cancer [26]. CTHRC1 promoted growth of pancreatic tumor and metastatic spread of cancer cells to distant organs. Besides, they also found overexpression of CTHRC1 in pancreatic cancer cells resulted in increased motility and adhesiveness. The similar effects of CTHRC1 in HCC and pancreatic cancer suggest that CTHRC1 is an important effector for tumor invasion. There are subtle differences between our results and theirs. We did not found CTHRC1 promotes growth of HCC cells. We found RhoA is the most important small GTPase for CTHRC1-mediated HCC invasion and they found Rac1 accounts for the invasiveness in pancreatic cancer. These observations indicate CTHRC1 may use similar but different mechanisms in different cells to promote tumor invasion.

Since CTHRC1 is a secretory protein highly expressed in cancers, it is likely to be aberrantly expressed in the serum of cancer patients and may be considered a promising tumor marker. Besides, the functional role of CTHRC1 in cancer cell migration and adhesion suggests that blocking CTHRC1, such with neutralizing antibody, may inhibit tumor metastasis and therefore be therapeutically beneficial.

## Supporting Information

Figure S1 **The ROC curve for CTHRC1 expression and patient death within four years in HCC patients who underwent surgical resection.**
(TIF)

Figure S2 **CTHRC1 expression did not affect the growth rate of Huh7 cells in NOD/SCID mice.** 2×10^6^ Huh7 cells were trypsinized, resuspended in serum-free DMEM, and injected subcutaneously into flanks. Animals were observed weekly for tumor development for 5 to 8 weeks. The final tumor weights at the time of animal sacrifice were recorded. The size and weights of the tumor masses were similar in CTHRC1 overexpressing and control groups.
(TIF)

Figure S3 **CTHRC1 is required for HA22T cell invasion through 3D collagen matrix.**
(TIF)
