# Supplementary figures and images for: Overexpression of CTHRC1 in Hepatocellular Carcinoma Promotes Tumor Invasion and Predicts Poor Prognosis
Source: PLoS One. 2013 Jul 29;8(7):e70324. doi: 10.1371/journal.pone.0070324 (PMC3726622; doi:10.1371/journal.pone.0070324)

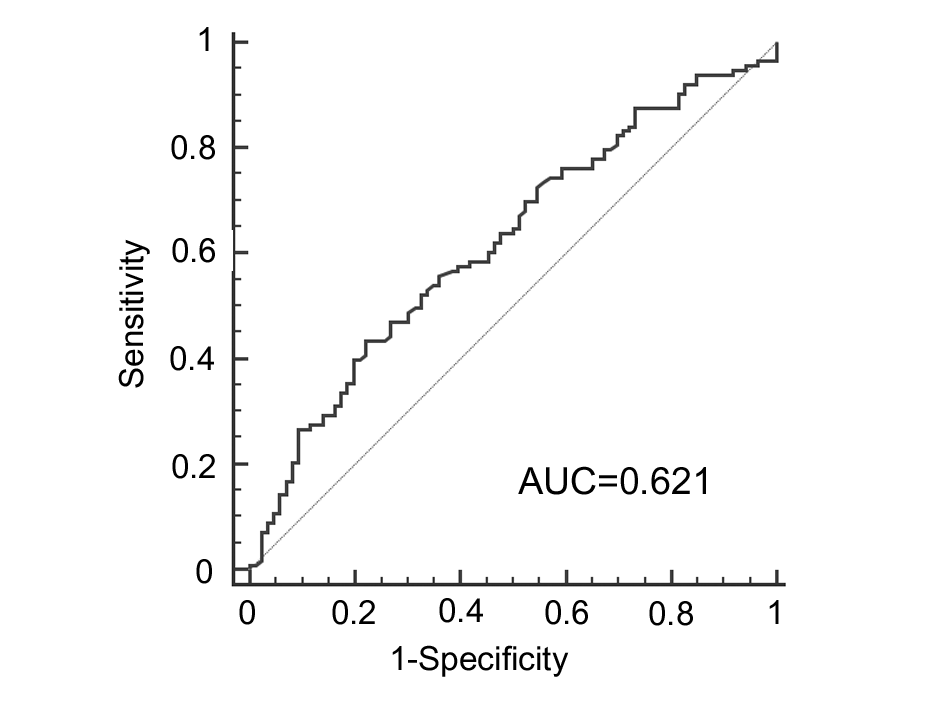

Supplement: Figure S1 — The ROC curve for CTHRC1 expression and patient death within four years in HCC patients who underwent surgical resection. (TIF) [file pone.0070324.s001.tif]

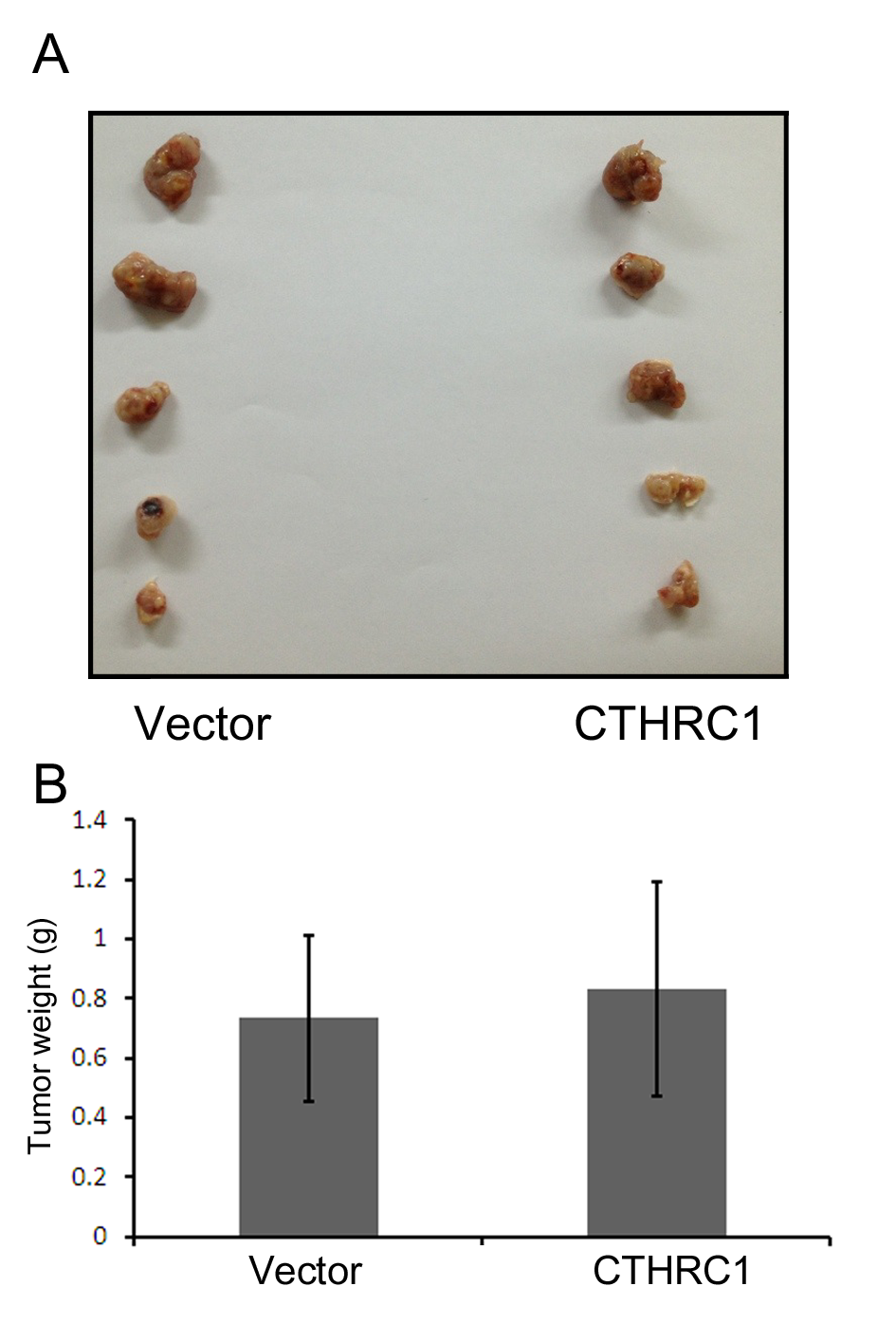

Supplement: Figure S2 — CTHRC1 expression did not affect the growth rate of Huh7 cells in NOD/SCID mice. 2×10^6^ Huh7 cells were trypsinized, resuspended in serum-free DMEM, and injected subcutaneously into flanks. Animals were observed weekly for tumor development for 5 to 8 weeks. The final tumor weights at the time of animal sacrifice were recorded. The size and weights of the tumor masses were similar in CTHRC1 overexpressing and control groups. (TIF) [file pone.0070324.s002.tif]

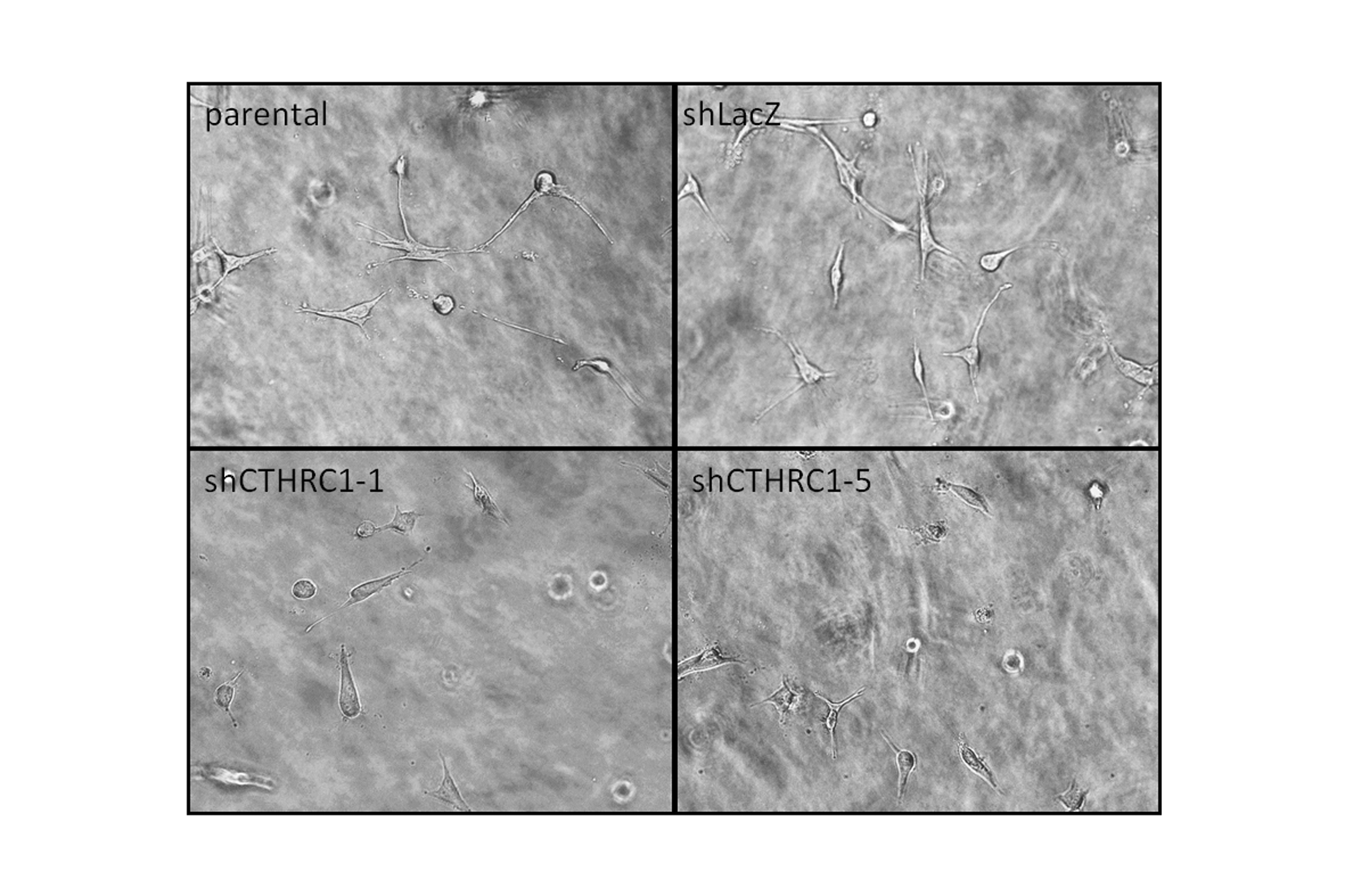

Supplement: Figure S3 — CTHRC1 is required for HA22T cell invasion through 3D collagen matrix. (TIF) [file pone.0070324.s003.tif]
